# Advances in Sensor-Based Technologies for Health, Aging, and Intelligent Care

**DOI:** 10.3390/s26103184

**Published:** 2026-05-18

**Authors:** Lili Liu, Antonio Miguel Cruz

**Affiliations:** 1 School of Public Health Sciences, Faculty of Health, University of Waterloo, Waterloo, ON N2L 3G1, Canada; lili.liu@uwaterloo.ca; 2 Department of Occupational Therapy, Faculty of Rehabilitation Medicine, University of Alberta, 8205-114 St, 2-64 Corbett Hall, Edmonton, AB T6G 2G4, Canada; 3 Research and Innovation, Glenrose Rehabilitation Hospital, 10230 111 Avenue NW, Edmonton, AB T5G 0B7, Canada

## 1. Introduction

The growing demand for scalable, privacy-preserving, and clinically meaningful health monitoring solutions has fueled rapid innovation in sensor technologies, machine learning, and intelligent systems. Aging populations, workforce shortages in care sectors, and the increasing emphasis on aging in place have further intensified the need for robust, unobtrusive approaches that support health assessment, risk detection, and intervention outside of traditional clinical environments.

This Special Issue brings together nine manuscripts that collectively advance the state of the art in sensor-based health monitoring, activity recognition, and intelligent care systems, with a strong focus on older adults, frailty, rehabilitation, and real-world deployment. The contributions span wearable, ambient, acoustic, radar-based, physiological, and neurophysiological sensing, highlighting how multimodal data and advanced analytics can support independent living, clinical decision-making, and user-centered care.

## 2. Overview of the Contributions

### 2.1. High-Accuracy and Ambient Monitoring for Frailty and Daily Activities

In Contribution 1, the authors present a high-accuracy smart home monitoring system that integrates ultra-wideband (UWB) indoor positioning with off-the-shelf Internet of Things devices to assess all five components of Fried’s Frailty Phenotype. Tested in a realistic simulated home environment, the system demonstrated strong concurrent validity against established clinical frailty scales, highlighting its potential for objective, continuous frailty assessment and early risk detection in aging-in-place contexts.

Building on indoor localization, Contribution 2 investigates automated detection of daily living activities using UWB sensors combined with inertial measurement units. Their work systematically evaluates anchor configurations and tag placement, demonstrating that the integration of precise positional data substantially improves activity recognition performance, achieving high classification accuracy in a mock condominium environment.

Complementing wearable and ambient approaches, in Contribution 3 the authors introduce an unsupervised learning framework for recognizing daily activities from household sounds. Using neural audio codecs and Dirichlet multinomial mixture models, the study shows that sound-based sensing—requiring minimal labeling—can effectively cluster daily activities, supporting the development of bespoke, privacy-conscious ambient assisted living systems.

### 2.2. Rehabilitation, Physical Function, and Work-Related Health

Addressing musculoskeletal health in occupational contexts, Contribution 4 examines the effects of different work–rest schedules on muscle fatigue during repetitive material handling tasks. Using wearable inertial sensors and electromyography, the study demonstrates that microbreaks and stretching routines significantly reduce fatigue without compromising productivity, offering evidence-based guidance for injury prevention in physically demanding jobs.

In the domain of rehabilitation and fall prevention, Contribution 5 reports findings from a randomized controlled trial evaluating a Nintendo™ Wii-based exercise program for older adults. The intervention resulted in significant improvements in balance, gait, flexibility, and lower limb strength, underscoring the value of interactive, game-based technologies in promoting physical function and reducing fall risk.

### 2.3. Radar-Based and Acoustic Sensing for Health Monitoring

Several contributions explore radar-based sensing as a privacy-preserving alternative to vision-based systems. Contribution 6 proposes an automated radar-based approach to measure step length in the home, a key yet underexplored gait parameter. Validated with frail older adults in both clinical and home environments, the system demonstrates strong agreement with gold-standard gait analysis tools, supporting its feasibility for continuous, real-world monitoring.

Extending radar applications to physiological monitoring, Contribution 7 presents a heart rate estimation method using MIMO FMCW radar and variational mode decomposition. By isolating heartbeat-related signal components and reconstructing robust features, the approach achieves high accuracy even in multi-subject scenarios, highlighting the potential of radar for unobtrusive vital sign monitoring.

From an acoustic perspective, Contribution 8 investigates voice-based pain level classification using lightweight convolutional neural network models. Their low-cost, sensor-assisted framework enables real-time classification of pain severity using verbal and nonverbal audio signals, demonstrating promise for intelligent care systems in both clinical and home settings.

### 2.4. Cognitive Health, Engagement, and Intelligent Interaction

Finally, Contribution 9 address cognitive health and engagement by exploring EEG-based monitoring of user engagement during cognitive gameplay. By linking EEG engagement indices with self-reported flow states and machine learning classification, the study provides evidence that neurophysiological signals can be used to assess and adapt cognitive interventions for both younger and older adults, supporting personalized and responsive rehabilitation technologies.

## 3. Concluding Remarks

Together, the papers in this Special Issue demonstrate the breadth and maturity of contemporary sensor-based health research. They illustrate how integrating advanced sensing technologies with data-driven analytics can enable meaningful assessment, monitoring, and intervention across diverse settings, from the workplace to the home. Importantly, many of these contributions emphasize real-world validation, user-centered design, and privacy-aware solutions—critical factors for successful translation into practice.

We hope this collection will inspire further interdisciplinary research and accelerate the adoption of intelligent sensor systems that promote health, independence, and quality of life across the lifespan.

## Data Availability

Not applicable.

## Abbreviations

The following abbreviations are used in this manuscript:

UWB	Ultra-Wideband
MIMO FMCW	Multiple-Input Multiple-Output Frequency-Modulated Continuous-Wave
EEG	Electro Encephalography

